# Indicators of Health-Related Quality of Life in Cats With Degenerative Joint Disease: Systematic Review and Proposal of a Conceptual Framework

**DOI:** 10.3389/fvets.2021.582148

**Published:** 2021-11-18

**Authors:** Gillian Yeowell, Danielle Burns, Francis Fatoye, Tadesse Gebrye, Andrea Wright, Kennedy Mwacalimba, Isaac Odeyemi

**Affiliations:** ^1^Department of Health Professions, Manchester Metropolitan University, Manchester, United Kingdom; ^2^Outcomes Research, International Center of Excellence, Zoetis, Dublin, Ireland; ^3^Outcomes Research, US Operations, Zoetis, Dublin, Ireland

**Keywords:** pain, health-related quality of life (HRQoL), cats, osteoarthritis (OA), conceptual framework

## Abstract

**Objectives:** The assessment of health-related quality of life (HRQoL) is becoming increasingly important in companion animals. This study describes a systematic review and development of a proposed conceptual framework to assess HRQoL in cats with osteoarthritis (OA).

**Methods:** The conceptual framework was developed according to published guidelines. A comprehensive search of the CAB Direct, Scopus, PubMed, and Web of Science databases was carried out for publications in English from inception to November 12, 2019. Search words used were “cat”, “feline”, “chronic pain”, “pain”, and “quality of life”. Publications were selected if they were full-text and peer-reviewed, based on primary data, and identified or measured behavioral symptoms of chronic musculoskeletal pain in cats. A systematic review was conducted according to Preferred Reporting Items for Systematic Reviews and Meta-Analyses (PRISMA) guidelines. A data extraction form was developed from categories identified in the literature review and piloted on a small number of studies to ascertain the appropriateness for relevant data extraction. Categories were then finalized, and key domains were identified. The domains were then synthesized to develop a conceptual framework.

**Results:** A total of 454 studies were identified, of which 14 met the eligibility criteria and were included in the meta-synthesis. All 14 were assessed to be of good quality. Seven domains related to HRQoL in cats with OA were thematically identified from the data: mobility, physical appearance, energy and vitality, mood, pain expression, sociability, and physical and mental wellbeing. The three main HRQoL domains were pain expression, mobility, and physical and mental wellbeing, which impacted all the others. Pain and mobility impacted all six other domains, with increased pain and decreased mobility negatively impacting physical appearance, energy and vitality, mood, sociability, and physical and mental wellbeing.

**Conclusions and Relevance:** This is the first study to develop an evidence-based conceptual framework for the assessment of HRQoL in cats with OA. The proposed conceptual framework suggests that effective management of chronic pain in cats may improve their overall HRQoL.

## Introduction

The feline medicalized population is an aging population, primarily due to increasing life expectancy in companion animals as a benefit of improved animal healthcare (1, 2). One condition associated with advancing years in cats is degenerative joint disease (DJD) or osteoarthritis (OA) (1, 3). Aging cats are more likely to show joint changes consistent with OA, and radiographic evidence of OA has been found in an estimated 90% of cats over the age of 12 (4).

OA is associated with chronic pain, which, in turn, can negatively impact health-related quality of life (HRQoL) (1). HRQoL is a multi-dimensional concept and includes factors such as pain and physical activity, and mental wellbeing (2, 5).

The assessment of HRQoL is becoming increasingly important in companion animals (2). HRQoL can be assessed using questionnaires developed with pet owners who then observe and report on the physical and emotional impacts of specific diseases like OA. However, a recent systematic review of quality of life (QoL) in cats by Doit et al. (6) highlights that within the literature, there is a lack of consensus regarding HRQoL in cats, and among the validated measures reviewed, one specific to assess OA has not been identified. Therefore, the first step in understanding the HRQoL impacts of OA is to develop a conceptual framework that proposes how chronic pain influences physical and mental wellbeing, based on a systematic literature review of QoL and OA in cats.

In general, the assessment of chronic pain in cats with OA is not straightforward as lameness is uncommon (3). Symptoms develop gradually and, as such, can be overlooked by pet owners and veterinarians (7–9). Furthermore, clinical detection relies primarily on owner reports, but subtle and non-specific symptoms may be incorrectly attributed to aging (10).

Therefore, there is need for research that identifies the factors that contribute to HRQoL in cats with OA. This will enable evaluations on how OA impacts HRQoL and, ultimately, the effective assessment of treatment interventions for cats with this condition. The aim of this study was to propose a conceptual framework for a tool that can assess HRQoL in cats with OA.

## Materials and Methods

The conceptual framework was developed according to published guidelines (11, 12). Development was undertaken in two phases. Phase 1 was a meta-synthesis that involved mapping the selected data sources, categorizing the data, and thematically identifying and naming key domains. Phase 2 was a synthesis of domains to develop the conceptual framework.

### Phase 1: Meta-Synthesis

Mapping of data sources was accomplished first by conducting a systematic review to identify the relevant literature. The review was conducted in accordance with the principles set out for reporting systematic reviews and meta-analyses, Preferred Reporting Items for Systematic Reviews and Meta-Analyses (PRISMA) guidelines (13).

#### Inclusion and Exclusion Criteria

Studies were eligible for inclusion in the meta-analysis if the following criteria were met: domestic cat was the main population; full-text, peer-reviewed journal article; articles based on primary data; measured/identified behavioral symptoms of chronic musculoskeletal pain; and articles were written in English. Articles were excluded if domestic cat was not the main population; article was not a full-text peer-reviewed journal article (e.g., conference paper, book chapter, citation only, or opinion piece); articles based on secondary data, e.g., reviews; cats were anesthetized; medical/physiological investigations of chronic MSK pain; article was not concerned with chronic MSK pain (i.e., acute and postoperative pain); focused on treatment/drug efficacy on chronic MSK pain; or was not written in English.

A comprehensive search strategy was developed to ensure that all relevant sources of data were identified. Searches were undertaken from inception to November 12, 2019 (date of the search) *via* the following electronic databases: CAB Direct, Scopus, PubMed, and Web of Science. The reference lists of all included studies, any relevant systematic reviews, and key background papers were scrutinized to identify any additional relevant studies. The keywords used were “cat”, “feline”, “chronic pain”, “pain”, and “quality of life”. These keywords were used in various combinations with Boolean operators “OR” and “AND” used to combine search terms.

#### Study Selection

**Figure 1:**
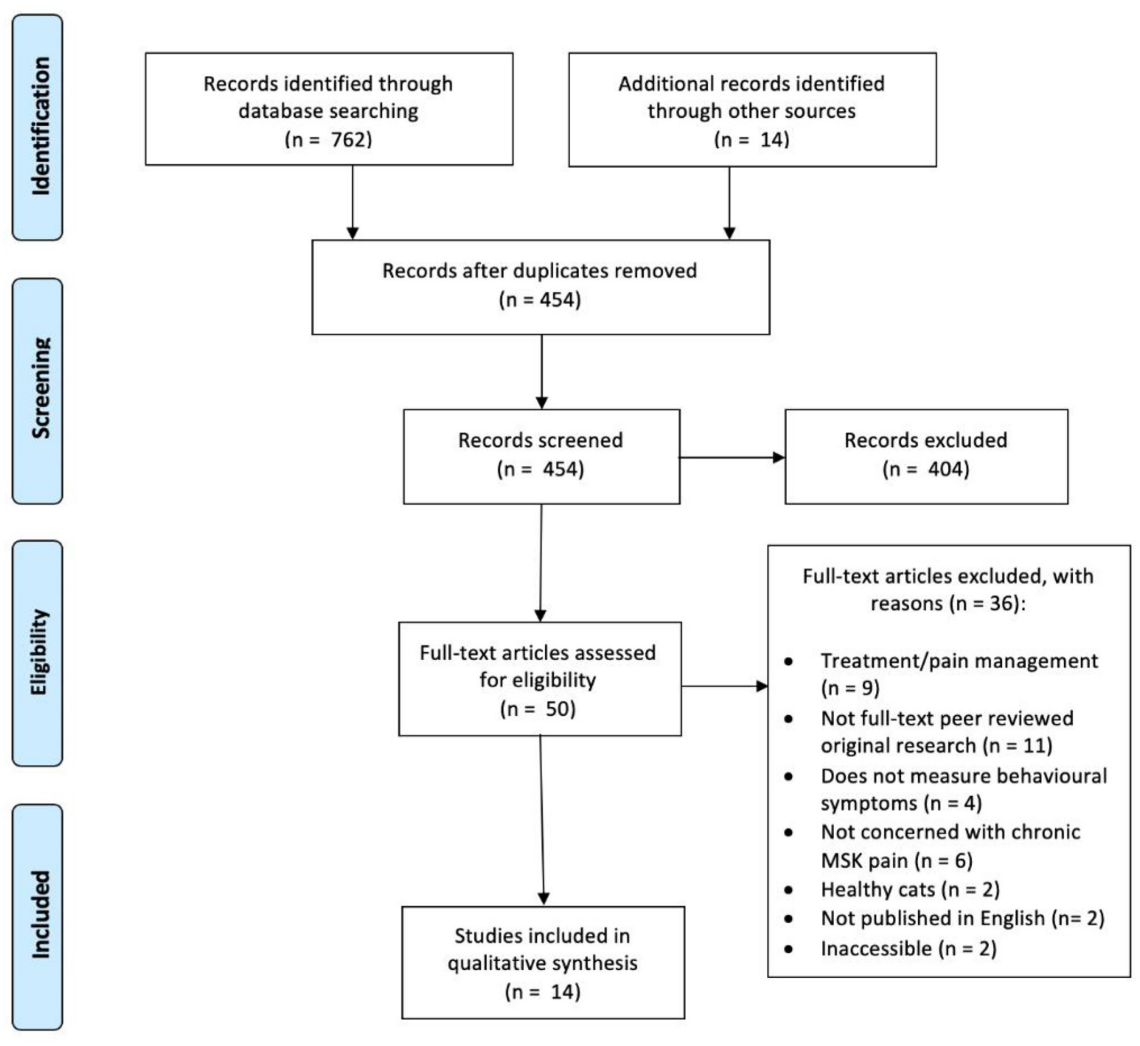
PRISMA flow diagram.

All searches were exported into EndNote Web (Thomas Reuter, CA, USA) bibliography software. Duplicates of articles were removed electronically and manually. Study selection was carried out in two stages. The first stage included title and abstract screening. To ensure that the relevant studies were identified and selected, two researchers independently screened titles and abstracts for eligibility based on the inclusion/exclusion criteria (DB and TG). Full-text articles were obtained and independently screened against the eligibility criteria (DB and TG). Manual searching of the reference lists was undertaken to identify any additional studies. Any disagreements in study selection between the researchers were resolved through discussion and consultation with two members of the project team (GY and FF). Stages in the study selection are illustrated in Figure 1 (PRISMA flow chart) (13).

#### Data Extraction

A data extraction form was developed from categories identified during the review of the literature (5, 14, 15). This was piloted on a small number of studies to ascertain the appropriateness for extracting relevant information. Piloting revealed some redundant categories (where no items were identified within them); categories that were conceptually similar and could be collapsed into one category; and the need for additional categories whereby items were not captured. The initial categories were mobility, emotion, energy, engagement, appetite, coat, eyes, fitness, sleep, and pain behavior. These were refined to mobility, physical appearance, energy and vitality, mood, pain expressions, sociability, and physical and mental wellbeing (Table 1). Data were extracted by one researcher (DB) and independently checked by a second researcher (TG). Two members of the project team (GY and FF) were available if there were disagreements, but were not required.

**Table 1:** Narrative of conceptual framework.

Health-related quality of life consisted of seven domains.
Three main domains were **pain expression, mobility**, and **physical and mental wellbeing**
**Pain expression impacted on**: Physical and mental wellbeing, mobility, sociability, mood, physical appearance, energy and vitality
**Pain expression was impacted by**: Mobility
**Physical and mental wellbeing impacted on**: Mood
**Physical and mental wellbeing was impacted by**: Pain, mobility, sociability, mood, energy and vitality
**Mobility impacted on**: Pain expression, physical and mental wellbeing, physical appearance, energy and vitality, sociability, mood
**Mobility was impacted by**: Pain, energy and vitality
**Mood impacted on**: Physical and mental wellbeing, energy and vitality, sociability
**Mood was impacted by**: Pain, mobility, physical and mental wellbeing, energy and vitality
**Physical appearance impacted on**: Physical and mental wellbeing
**Physical appearance was impacted by**: Pain, mobility, energy and vitality
**Energy and vitality impacted on**: Physical and mental wellbeing, mobility
**Energy and vitality was impacted by**: Pain, mobility, mood
**Sociability impacted on**: Physical and mental wellbeing
**Sociability was impacted by**: Pain, mobility, mood, energy and vitality

#### Quality Assessment

The methodological quality of the identified studies was critically appraised using the criteria listed in the ARRIVE guideline for reporting animal research (16) and the veterinary (STROBE-Vet) statement (17). The ARRIVE guideline contains 20 criteria and can be used to evaluate studies of bioscience research using laboratory animals and comparative experiments, whereas the Veterinary (STROBE-Vet) statement can be applied for observational studies. A score of 1 was assigned if they fulfilled the requirement of reporting for the item completely, and 0 for not reporting. The maximum score for an article that reported all information was 24 for the STROBE-Vet statement and 20 for the ARRIVE guideline. A cutoff point score of 50% was used to assess the quality of the included studies (18). Studies were determined to be of “good quality” if they scored ≥50%, whereas studies were considered “poor quality” studies if they scored <50% (18). The final step in Phase 1 was to categorize the data and confirm the key domains.

### Phase 2: Synthesis of Domains to Develop Conceptual Framework

**Figure 2:**
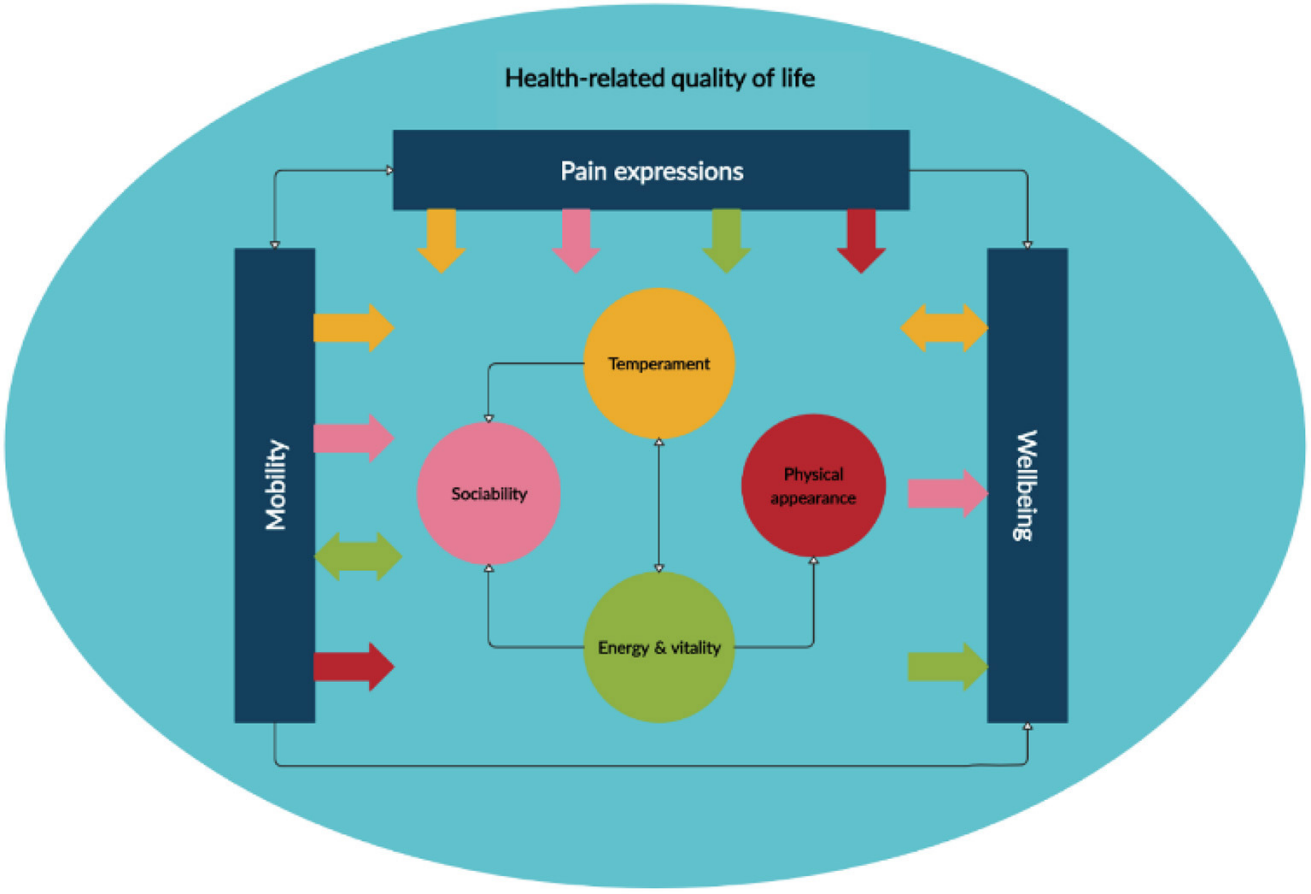
Conceptual framework.

In this phase, a thematic analysis framework, as described by Braun and Clarke (19), was used to inductively analyze the extracted data (Supplementary File 1) to identify HRQoL domains. The data were synthesized into a narrative under each domain to develop the conceptual framework (Figure 2). The approach to narrative synthesis has recently been described elsewhere (20). The proposed conceptual framework underwent preliminary review with three stakeholders.

### Ethics

Ethical approval was obtained from the University Faculty Health, Psychology and Social Care Research Ethics and Governance Committee (UK): Reference Number: 10368.

## Results

A total of 776 studies were identified. After removing duplicates, and applying the inclusion criteria and abstract screening, 14 studies met the inclusion criteria (Figure 1) and were included in the meta-synthesis (Supplementary Material Table 2).

Of the 14 studies included, six studies were from the USA (15, 21–25), 4 were from the UK (1, 5, 26, 27), three studies were from Canada (10, 28, 29), and there was one study from the Netherlands (30). Eleven studies were observational and three were comparative studies. The total number of cats included was 1,168, and the number of cat owners was 324.

The scores of the methodological quality assessment are presented (Supplementary Materials). Eight studies (1, 10, 15, 21–24, 29) achieved more than 70% of the checklist criteria. The remaining six studies (5, 25–28, 30) scored between 50 and 70% of the criteria. All included studies scored ≥50% and, as such, met the criteria to be considered good quality (18).

### Categorizing the Data and Identifying and Naming Key Domains

**Figure 3:**
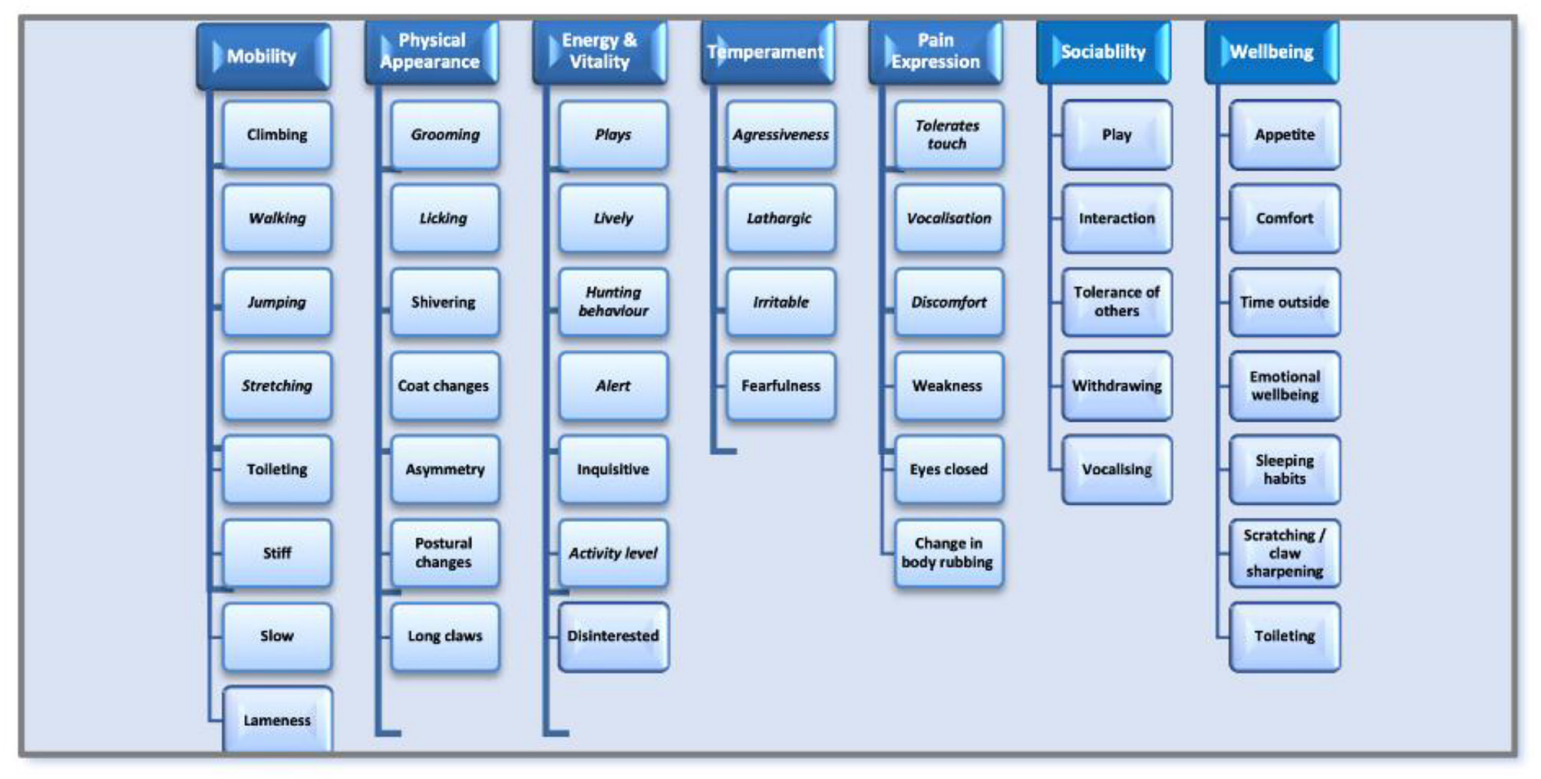
Quality-of-life domains.

From the data extracted, data were categorized, and key domains were confirmed (Figure 3). Seven domains were identified from the data: mobility, physical appearance, energy and vitality, mood, pain expression, sociability, and physical and mental wellbeing. The items that formed these domains are presented in Figure 3.

### Synthesis of Domains to Develop Conceptual Framework

Key domains were synthesized to develop the conceptual framework (Figure 2). The three main domains in terms of frequency and impact were pain expression, mobility, and physical and mental wellbeing. A narrative of how these domains interact is presented in Table 1.

Initial validation of the conceptual framework was undertaken. The conceptual framework was shared with three key stakeholders: a veterinarian practicing in companion animals (UK based), an owner of an aged cat (15 years) (UK based), and a representative from a pharma company specializing in animal pharma (USA based), to obtain their views and feedback. They confirmed that all domains were relevant and their relationship was appropriate. No changes were requested. All participants confirmed the conceptual framework accurately and wholly reflected the domains that impact HRQoL in cats with OA. A subsequent qualitative concept elicitation study with key informants including veterinarians and cat owners will help us confirm that the HRQoL domains and areas of overlap in our model reflect real-world experiences. Thus, our framework may be considered preliminary until we can further validate it with qualitative evidence.

## Discussion

To the authors' knowledge, this is the first study to develop an evidence-based conceptual framework for the assessment of HRQoL in cats with OA. To achieve this, a systematic review was undertaken to identify the relevant literature to develop the conceptual framework. Fourteen articles were identified, all of which were assessed as good quality. From the data extracted, seven domains were identified in relation to HRQoL in cats with OA. These domains were mobility, physical appearance, energy and vitality, mood, pain expression, sociability, and physical and mental wellbeing. Of these, three domains, pain expression, mobility, and physical and mental wellbeing, were identified as being more important to HRQoL in cats with OA. Feline physical and mental wellbeing was impacted by five of the six domains. This preliminary conceptual framework was found to be a valid representation of HRQoL in cats with OA. A similar conceptual framework development approach has recently been described in human and animal health (20, 31).

There is evidence that the relationship between pain and QoL is complex (32). This study adds to this evidence in relation to chronic pain and HRQoL in cats. Pain and mobility were identified as impacting on all other domains, with increased pain and decreased mobility negatively impacting physical appearance, energy and vitality, mood, sociability, and physical and mental wellbeing. Pain is only one of the main domains that impacts HRQoL. Other domains can also negatively impact HRQoL. However, this conceptual framework suggests that effectively managing chronic pain in cats may improve their HRQoL. In addition, if pain is reduced, the other domains of HRQoL, for example, mobility and physical and mental wellbeing, may also improve.

There is a lack of research that has investigated HRQoL with OA in cats. From the systematic review, only 14 studies were identified. Of these, only one study (15) looked at HRQoL and OA. Benito et al. (15) undertook a cross-sectional study to identify items considered important for their cat's QoL by owners of cats with OA. However, the study focused on QoL in relation to mobility. In addition, 95% of the cat owners surveyed indicated that their cats were indoor cats. This limits the generalizability of this study to the wider population of cats.

The remaining studies focused on pain and DJD or OA (10, 21, 22, 25, 29), pain and behavior change (1, 27) spondylosis and behavior change (30), clinical signs and OA (26, 28) activity and painful OA (24) and the development of a generic instrument to measure HRQoL in a range of chronic diseases (5).

There were some limitations to this study. Only articles published in English were included; therefore, there is the possibility that some further data were available to inform the conceptual framework. However, preliminary review of the conceptual framework suggests that it was a comprehensive and accurate reflection of the domains that impact HRQoL in cats with DJD. A subsequent qualitative concept elicitation study with key informants including veterinarians and cat owners will help us confirm that the HRQoL domains and areas of overlap in our model reflect real-world experiences. Then, the development of an evidence-based tool to assess HRQoL in cats with OA can be undertaken.

## Conclusion

This study has developed a conceptual framework for a tool that can assess HRQoL in cats with OA. Seven domains were identified in relation to HRQoL in cats with OA: mobility, physical appearance, energy and vitality, mood, pain expression, sociability, and physical and mental wellbeing. The three main domains were pain expression, mobility, and physical and mental wellbeing. This conceptual framework suggests that effective management of chronic pain in cats with OA may improve their HRQoL. However, other domains can also negatively impact HRQoL. The findings of this study can be used to inform the development of an evidence-based tool to assess HRQoL in cats with OA.

## Data Availability Statement

The original contributions presented in the study are included in the article/Supplementary Material, further inquiries can be directed to the corresponding authors.

## Author Contributions

GY: study design, data analysis, and manuscript development. DB and TG: data collection, data analysis, and final report preparation. FF: study design, data collection, data analysis, and manuscript development. AW: study design, quality assurance, and manuscript preparation. KM: quality assurance and manuscript preparation. IO: study design and manuscript development.

## Funding

The authors declare that this study was funded by Zoetis.

## Conflict of Interest

AW, KM, and IO work for Zoetis that funded the developed the conceptual framework. The remaining authors declare that the research was conducted in the absence of any commercial or financial relationships that could be construed as a potential conflict of interest.

## Publisher's Note

All claims expressed in this article are solely those of the authors and do not necessarily represent those of their affiliated organizations, or those of the publisher, the editors and the reviewers. Any product that may be evaluated in this article, or claim that may be made by its manufacturer, is not guaranteed or endorsed by the publisher.
