# Supplementary material for: Indicators of Health-Related Quality of Life in Cats With Degenerative Joint Disease: Systematic Review and Proposal of a Conceptual Framework
Source: Front Vet Sci. 2021 Nov 18;8:582148. doi: 10.3389/fvets.2021.582148 (PMC8636455; doi:10.3389/fvets.2021.582148)
Supplement: Supplementary file 1 [file Table_1.docx]

**Supplementary File 1. Data extraction to show quality of life indicators**

| **Author**  **Year, origin** | **Study Design**  **Sample (n)** | **Health-related quality of life indicators** | | | | | | | |
| --- | --- | --- | --- | --- | --- | --- | --- | --- | --- |
|  |  | | **Mobility** | **Physical appearance** | **Energy & Vitality** | **Mood** | **Pain expressions** | **Sociability** | **Physical & Mental Wellbeing** |
| Benito et al. 2012  USA | Prospective, observational study  Cats total (n = 166)  [n = 89 cats with degenerative joint disease] | | - Active transition (moving from point A to B) - AA | - Grooming-scratching (AA/IN) | - Playing-hunting (AA) - Resting-observing (IN) | - *Not measured/ reported* | - *Not measured/ reported* | - Social activities (not specified) | - Eating-drinking (IN) |
| Benito et al. 2013a  USA | Questionnaire psychometric testing  Cats total (n = 32)  [normal n = 13, degenerative joint disease n = 19] | | - Climb up stairs or steps? - Go down stairs or steps? - Walk and/or move easily? - Jump up (how well or how easily)? - Jump up to kitchen counter height in one try? - Jump down (how well or how easily)? - Getting up from a resting position? - Lie and/or sit down? - Stretch? - Use the litter box (get in and out, squat, cover waste?) - Run? | Groom himself or herself? | - Play with toys and/ or chase objects? - Overall activity? | *Not measured /reported* | - Tolerate being touched and/or held? - Pain over the last week? (no to severe pain) - Pain today? (no to severe pain) | - Play and interact with other pets? - Interact with you and family members? | - Eat? - Overall QoL? |
| Benito et al. 2013b  USA | Questionnaire psychometric testing  Cats (n = 25) with degenerative joint disease | | - Climb up stairs or steps? - Go down stairs or steps? - Walk and/or move easily? - Jump up (how well or how easily)? - Jump up to kitchen counter height in one try? - Jump down (how well or how easily)? - Getting up from a resting position? - Lie and/or sit down? - Stretch? - Use the litter box (get in and out, squat, cover waste?) - Run? | - Groom himself or herself? | - Play with toys and/or chase objects? - Overall activity? | *Not measured/ reported* | - Tolerate being touched and/or held? - Pain over the last week? (no to severe pain) - Pain today? (no to severe pain) | - Play and interact with other pets? - Interact with you and family members? | - Eat? - Overall QoL? |
| Bennett & Morton  2009  UK | Questionnaire psychometric methodology  Cats (n = 23) with chronic musculoskeletal pain | | - Mobility - Jumping up or down - Refusing or hesitating to jump up or down - Less agile on stairs - No longer attempting to reach high spots - Size/height of jump, up or down - Makes smaller jumps e.g. takes smaller steps to reach high spots - Frequency of jumps e.g. jumps onto high surfaces less than before - Gracefulness - Movements less graceful than before - Becoming stiff or creaky - Changes in toileting - Changes in location e.g. reluctant/refusing to go outside or use litter tray - Difficulties using litter tray e.g. missing tray sometimes/often | - Coat condition - Coat matted or ‘scurfy’, generally or in one particular place - Observed grooming behaviour less frequent or of shorter duration - Claws overgrown or catching on carpets or clicking on hard floors - Grooming habits | - Playing - Playing less - No longer instigating play - More difficult to tempt to play - Hunting - Hunting less than before - Activity levels - Quieter | - Temperament (demeanour) | - *Not measured /reported* | - Tolerance to owner or other animals - Less keen to interact - Grumpy on contact with other cats - Grumpy on contact with other animals including owner - Not seeking/avoiding contact with owner - General attitude - Spending more time alone - Not seeking/avoiding contact with other cats or other animals | - Scratching - Sharpens claws less frequently - Change of location/height where scratching occurs - Sleeping habits - Sleeping or resting more - Lying in the same spot for a long time, not moving often - Changes in resting place |
| Clarke & Bennett 2006  UK | Prospective cohort study  Cats (n = 28) with osteoarthritis | | - Unwilling to jump - Reduced height of jump - Stiff gait - Lameness | - *Not measured /reported* | - General demeanour (activity level) – normal to totally disinterested | - *Not measured /reported* | - Vocalises if handled - Aggressive if handled - Resents handling | - Seeks seclusion | - Food intake – unchanged to severely decreased and increased |
| Gruen et al. 2017  USA | Clinical trial  Cats total  (n = 98)  [Cats with (n = 83) and without (n = 15) degenerative joint disease] | | *Not measured /reported* | *Not measured /reported* | Activity levels: intensity and profile | *Not measured /reported* | *Not measured /reported* | - Total pain scores | *Not measured /reported* |
| Klinck et al. 2012  Canada | Cross-sectional interview survey design  Cat owner surveys (n = 50) | | - Reduction or cessation in jumping & stair use Changes in jumping and stair use such as hesitation, stumbling/falling, doing several small jumps instead of one large jump, doing a few stairs rather than entire flight - Eliminating outside of litter box due to urgency/reluctance to navigate stairs - Difficulty manoeuvring in box - Clumsiness - Gait changes - Limping or stiffness (more severe after rest) - Changes in limb carriage or appearance | - Decreases in grooming - Decreases and increase in grooming of particular areas, - Coat changes - Changes of posture - Change in preferred positions - Asymmetry or other posture abnormalities e.g. hunched posture, holding a paw up - Claws that were longer, brittle or more dull | - Increased time resting - Decreased playfulness/ tolerance - Decreased activity level - Changes to play and hunting - Reductions or cessation - Playing in recumbent position - No longer following birds in the window   Other changes   - Less adventurous - Lying down/getting up slowly or with difficulty | - Poorer mood - Fearfulness - Altered overall character or frequency of vocalisations | - Reacted to being picked up or touched in specific areas Asking for help e.g. vocalising, staring, tapping with paw - Reduction in head or body rubbing Increase in head/body rubbing - Change in target of head/body rubbing - Meowing from other parts of house or when moving about | - Increased or decreased friendliness to strangers - Increased or decreased interactions with family members e.g., sleeping with them more/less - Increased vocalising to owner - Decreased meowing to be let out - Changes in interaction with household animals - Increased frequency of being chased/picked on - Hiding | - Less time spent outside - Seeking heat/sun Reduction in appetite - Increase in appetite Variability in diet - Decreases or changes to claw-sharpening such as on horizontal rather than vertical surfaces - Worsening signs in damp weather |
| Klinck et al. 2018a  Canada | Questionnaire design and psychometric testing via randomised crossover study  Mi-CAT©  Pilot (n = 11 cats with and without osteoarthritis)  Crossover study (n = 54 cats with osteoarthritis) | | - My cat moves smoothly and gracefully - My cat can jump UP 3 feet or more to reach high places to rest in, play on, or investigate - My cat can jump DOWN 3 feet or more - My cat easily runs UP stairs - My cat easily runs DOWN stairs - My cat regularly stretches by extending both front feet forward and then both back feet out behind - My cat can easily scratch their head or neck with either hind foot - My cat moves clumsily or awkwardly - My cat moves stiffly or limps - My cat gets up stiffly after resting - My cat seems to hesitate or to avoid jumping UP - My cat seems to hesitate or to avoid jumping DOWN - My cat can’t/won’t jump UP more than 1-1½ feet - My cat can’t/won’t jump DOWN more than 1-1½ feet - My cat has trouble getting to/into, or out of the litter box, or gets urine or stool outside the box - My cat climbs vertical surfaces (e.g cat tower, furniture, trees) - My cat prefers several small hops to one bigger jump UP - My cat prefers several small hops to one bigger jump DOWN - My cat hesitates/pauses going UP stairs or takes them slowly/one at a time - My cat hesitates/pauses going DOWN stairs or takes them slowly/one at a time - My cat asks to be lifted or carried up/down the stairs or to/from elevated locations (e.g., window, table, bed) - My cat runs on flat surfaces | - My cat looks hunched or crouched when standing or walking - My cat tends to have their weight shifted off one (or more) particular legs or puts a leg out to the side when walking, standing, sitting, or lying - My cat can groom their whole body easily - My cat’s fur seems dull/flaky/untidy - My cat doesn’t wash themselves well or often - My cat’s claws are long/dull, or get caught in things | - My cat sneaks/tries to sneak through doors to get outside or into off-limits parts of the house - My cat doesn’t like to play - My cat investigates and plays with new objects or furniture - My cat steals/attempts to steal people food or other food that isn’t in a pet dish - My cat leaps into mid-air when playing | - *Not measured /reported* | - *Not measured /reported* | - My cat lies on or against family members or other pets - My cat wants to play with family members/other pets - My cat enjoys being picked up or held | - My cat stretches up to sharpen their claws on, or to paw at (if declawed), vertical surfaces - My cat sharpens their claws on, or paws at, horizontal surfaces |
| Klinck et al. 2018b  Canada | Questionnaire design and testing (video analysis and therapeutic trials)  Mi-CAT (V)  **Video analysis** (n = 10 cats) [n=6 with and n = 4 without osteoarthritis]  **Therapeutic trials** (n = 12 - 20 cats)   - Phase I total cats n = 12 [n = 7 with OA; n = 5 non-OA] - Phase II total cats n = 20 [n = 15 with OA; n = 5 non-OA] - Phase III total cats n = 19 [n = 13 with OA; n = 6 non-OA] | | ***Gait***   - Appears to move slowly - Lateral movements of spine at walk - Forelimb stiffness/lameness - Forelimb circumduction - Forelimb joints – reduced range of motion - Hind limb stiffness/limping - Hind limb circumduction - Hind limb joints – reduced range of motion   ***Willingness and Ease of Horizontal Movements (scoring change)***   - How willing to walk? - How willing to trot? - How willing to gallop? - Decreased locomotion towards end of assessment - When encouraged to move about, cat usually: gallops/trots, trots/walks, walks? - Reverses direction by pivoting quickly/smoothly on hind legs - Sits down (NOT a crouch) without encouragement - Goes into a sit (NOT a crouch) from standing:   ***Jumping***   - Prefers to stand on hind limbs to reach for treats - Requires encouragement to jump up - Hesitates when jumping up - Uses front feet to aid jump up - Seems to hesitate/prepare to jump DOWN - Hind feet land heavily (audibly/visibly) when jumping down   ***Scoring change***   - Slinks (with gliding steps) rather than jumping down - Hind feet land simultaneously when jumping DOWN   ***Other behaviours***   - Tends to move along the edge of the room (wall) - Stretches hind legs by lifting and extending backward one at a time   ***Global Distance Examination – General Lameness Score***   - Scored from 0 (No lameness) to 10 (worst possible OA-related lameness) | - ***Body Posture – Back*** - Back has a dip just caudal to shoulders - Back has a T-L/lumbar/L-S hump - Front end lower than hind - Hind end lower than front - ***Body Posture – Forelimbs*** - Asymmetry (right to left) - Uneven weight distribution (right to left) - Limb abduction - External rotation (toe(s) turned out) - Increased forelimb flexion - Increased forelimb extension - ***Body Posture – Hind limbs*** - Asymmetry (right to left) - Limb abduction - Base wide appearance - External rotation (hock(s) turned in) - Stands with hind limbs held far forward under body - Increased hind limb flexion - Increased hind limb extension | - *Not measured /reported* | - *Not measured /reported* | - *Not measured /reported* | - *Not measured /reported* | - *Not measured /reported* |
| Kranenburg et al.  2012  Netherlands | Cross-sectional questionnaire  Group 1  Radiographic prevalence of spondylosis (n = 402 cats)  Group 2  Cat owner surveys (n = 100) | | - Change in jumping on **AND** off objects - Has the height of jumping on **AND** off decreased? - Change in jumping high jumps - Change in walking upstairs **AND** downstairs? - Change in stiffness Change in accessibility of cat’s favourite places - Elimination directly over edge of box - Change in elimination directly over the edge of the litter box - Lameness in past? In present? - Ever hit by a car? | - Change in number of grooming moments - Change in time spent grooming | *Not measured/reported* | - Change in aggressiveness - Change in satisfaction | Change in acceptance of being petted | - Change in greeting people - Change in inter-cat socialisation | - Indoor or outdoor cat or both? - Time spent outside? |
| Lascelles et al.  2007  USA | Observational study  Evaluation of subjective assessments and activity monitor    Cats (n = 13) with arthritis | | Cat activity was assessed by using client-specific outcome measures [CSOM] and accelerometer.  Owners indicate how problematic activities are for their cat compared to when their cat did not have arthritis. | *Not measured /reported* | *Not measured /reported* | *Not measured /reported* | *Not measured /reported* | *Not measured /reported* | *Not measured /reported* |
| Merola & Mills  2016  UK | Delphi methodology  4 rounds of consultation with 19 feline medicinal experts | | - Difficulty to jump (S) - Reluctant to move (S) - Abnormal gait (S) - Lameness (S) | - Hunched up posture (S) - Lower head posture (S) - Overgrooming (I) - Absence of grooming (S) - Licking a particular body region (S) - Eat rotated (I) - Ear downward (I) - Ear flattener – sign of fear (I) - Shifting of weight (S) - Tongue showing (I) - Trembling/shivering – rare in cats (I) - Body tense (I) | - Playing less (S) - Sitting more often (I) - Overall activity decreased (S) | - General mood (e.g. tendency to be irritable due to episode of pain) (S) - Temperament (more consistent to chronic pain) (S) - Hissing – related to temperament (I) | - Rolled up (I) - Standing longer than usual (I) - Half blink (I) - Reaction to palpation (S) - Growling (useful if new behaviour) (S) - Groaning (not reliable) (S) - Eyes closed (other possible causes) (S) - Lying on its side (I) - Crouching (I) - Meowing (I) - Crying (I) - Less rubbing on objects (I) - Teeth grinding – rare in cats (I) - Spitting (I) - Purring (I) | - Withdraw/hiding (S) - Less rubbing toward people (S) - Seeking contact with a person (I) - Trying to scratch someone (I) - Trying to bite someone (I) - Hiding down in the litter box (I) - Escaping when trying to catch, important if change from normal (I) | - House soiling (I) - Reduction urination (I) - Appetite decrease (S) - Change in form of feeding behaviour (knowledge of prior feeding habits needed) (S) - Sleeping more (I) - Sleeping less (I) - Scratching less (I) |
| Noble et al. 2019  UK | Questionnaire design & psychometric testing  -Interview cat owners n = 18  -Field Test 1  n = 71 single owners  n = 30 healthy cats  n = 41 sick cats  -Field Test 2  n = 94 single owners  n = 26 healthy cats  n = 58 sick cats | | - Unsteady - Jumping or climbing up/down as usual - Stiff - Slow | - *Not measured reported* | - Active - Energetic - Lively - Playful - Shows hunting behaviour - Alert - Exploring - Inquisitive - Vitality | - Content - Enjoying the things he normally does - Feeling himself - Happy - Lethargic | - Sore | - *Not measured/ reported* | - Comfortable - Uncomfortable - Emotional wellbeing |
| Zamprogno et al.  2010  USA | Questionnaire design and evaluation  Part I  Cats (n = 100) [93 had radiological evidence of degenerative joint disease]  Part II  Interviews with cat owners (n = 30). Plus 3 focus groups with 5 cat owners in each group (total n = 15)  Field testing with final 15 items derived from interview/focus groups | | - Difficulty jumping Difficulty using the litterbox - Ability to move around - Ability to use litterbox - Ability to climb stairs - Ability to jump Difficulty rising from a resting position | - Lack of grooming - Ability to groom themselves Changes in posture | - Being active - Decreased activity - Decreased movement - Not playing with toys - Restlessness - Ability to play with toys - Absence of so-called bursts of energy - Ability to chase objects | - Quiet or lethargic - Behavioural abnormalities | - Vocalisation - Time of onset after long rest - Does not like to be touched - Changes in the position that cat lies in - Discomfort being held - Grunting when jumping down - Weakness | - Decreased interaction with owner and other pets - Hiding - Increased interaction, owners and pets - Willingness to interact - Being given affection - Having other pets to interact with | - Increased sleeping - Ability to sleep well - Being comfortable - A safe, quiet stimulating place to live - Being fed - Normal routine - Being healthy - Having plenty of water - Being brushed - Being owned - Being kept indoors - Decreased appetite - Ability and willingness to eat - Ability and willingness to drink - Willingness to go outside |

**KEY: QoL** = Quality of life**; (S)** = denotes sufficient to infer pain, **(I)** = denotes insufficient to infer pain but possibly present in high/low levels of pain**; (AA)** = involving motion, **(IN)** = not involving motion, **(IA)** = inactive with implied activity

Table 2 Quality Assessment: The veterinary (STROBE-Vet) statement for observational studies (Sargeant et al. 2016)

| **Item** | **Benito et al. 2013** | **Benito et al. 2012** | **Benito et al. 2013** | **Bennett & Morton 2009** | **Clarke & Bennett 2006** | **Klinck et al. 2012** | **Merola & Mills 2016** | **Lascelles et al. 2007** | **Kranenburg et al. 2012** | **Noble et al. 2019** | **Zamprogno et al. 2010** |
| --- | --- | --- | --- | --- | --- | --- | --- | --- | --- | --- | --- |
| Indicate that the study was an observational study and, if applicable, use a common study design term/ Indicate why the study was conducted, the design, the results, the limitations, and the relevance of the findings. | Yes | Yes | Yes | Yes | Yes | Yes | Yes | Yes | Yes | Yes | Yes |
| The study stated specific objectives, including any primary or secondary prespecified hypotheses or their absence | Yes | Yes | Yes | Yes | Yes | No | Yes | Yes | Yes | Yes | Yes |
| The study ensured that the level of organisation is clear for each objective and hypothesis | Yes | Yes | Yes | Yes | Yes | Yes | Yes | Yes | Yes | Yes | Yes |
| Presented key elements of study design early in the paper | Yes | Yes | Yes | Yes | Yes | Yes | Yes | Yes | Yes | Yes | Yes |
| Describe the setting, locations, and relevant dates, including periods of recruitment, exposure, follow-up, and data collection; If applicable, include information at each level of organization. | Yes | Yes | Yes | Yes | Yes | Yes | Yes | Yes | Yes | Yes | Yes |
| Describe the eligibility criteria for the owners/ managers and for the animals, at each relevant level of organization; describe the sources and methods of selection for the owners/managers and for the animals, at each relevant level of organization; describe the method of follow-up; For matched studies, describe matching criteria and the number of matched individuals per subject (e.g, number of controls per case) | Yes | Yes | Yes | Yes | Yes | Yes | No | Yes | Yes | Yes | Yes |
| Clearly deﬁne all outcomes, exposures, predictors, potential confounders, and eﬀect modiﬁers. If applicable, give diagnostic criteria; describe the level of organization at which each variable was measured; for hypothesis-driven studies, the putative causal structure among variables should be described (a diagram is strongly encouraged). | No | Yes | Yes | Yes | No | Yes | No | Yes | Yes | Yes | Yes |
| For each variable of interest, give sources of data and details of methods of assessment (measurement). If applicable, describe comparability of assessment methods among groups and over time; If a questionnaire was used to collect data, describe its development, validation, and administration; describe whether or not individuals involved in data collection were blinded, when applicable. | Yes | Yes | Yes | Yes | Yes | No | No | Yes | Yes | Yes | Yes |
| Describe any eﬀorts to address potential sources of bias due to confounding, selection, or information bias. | No | No | No | No | No | No | No | No | No | No | No |
| Describe how the study size was arrived at for each relevant level of organization; describe how non-independence of measurements was incorporated into sample-size considerations, if applicable; If a formal sample-size calculation was used, describe the parameters, assumptions, and methods that were used, including a justiﬁcation for the eﬀect size selected. | No | No | No | No | No | No | No | No | No | No | No |
| Explain how quantitative variables were handled in the analyses. If applicable, describe which groupings were chosen, and why | No | Yes | Yes | Yes | Yes | No | No | Yes | Yes | No | No |
| Describe all statistical methods for each objective, at a level of detail sufficient for a knowledgeable reader to replicate the methods. Include a description of the approaches to variable selection, control of confounding, and methods used to control for non-independence of observations; describe the rationale for examining subgroups and interactions and the methods used; Explain how missing data were addressed; ff applicable, describe the analytical approach to loss to follow-up, matching, complex sampling, and multiplicity of analyses; Describe any methods used to assess the robustness of the analyses (e.g, sensitivity analyses or quantitative bias assessment). | Yes | Yes | Yes | Yes | Yes | No | Yes | Yes | Yes | No | No |
| Report the numbers of owners/managers and animals at each stage of study and at each relevant level of organization - e.g, numbers eligible, included in the study, completing follow-up, and analyzed; Give reasons for non-participation at each stage and at each relevant level of organization; Consider use of a ﬂow diagram, a diagram of the organizational structure or both. | Yes | Yes | Yes | Yes | Yes | Yes | Yes | Yes | Yes | Yes | Yes |
| Give characteristics of study participants (e.g, demographic, clinical, social) and information on exposures and potential confounders by group and level of organization, if applicable; Indicate number of participants with missing data for each variable of interest and at all relevant levels of organization; summarize follow-up time (e.g, average and total amount), if appropriate to the study design | Yes | Yes | Yes | Yes | Yes | Yes | Yes | Yes | Yes | Yes | Yes |
| Report outcomes as appropriate for the study design and summarize at all relevant levels of organization; for proportions and rates, report the numerator and denominator; For continuous outcomes, report the number of observations and a measure of variability. | Yes | Yes | Yes | Yes | Yes | Yes | Yes | Yes | Yes | Yes | Yes |
| Give unadjusted estimates and, if applicable, adjusted estimates and their precision (e.g, 95% conﬁdence interval). Make clear which confounders and interactions were adjusted. Report all relevant parameters that were part of the model; Report category boundaries when continuous variables were categorized; If relevant, consider translating estimates of relative risk into absolute risk for a meaningful time period; | Yes | Yes | No | No | No | No | No | No | No | No | Yes |
| Report other analyses done, such as sensitivity/ robustness analysis and analysis of subgroups | No | No | No | No | No | No | No | No | No | No | No |
| Summarize key results with reference to study objectives | Yes | Yes | Yes | Yes | Yes | Yes | Yes | Yes | Yes | Yes | Yes |
| Discuss strengths and limitations of the study, taking into account sources of potential bias or imprecision. Discuss both direction and magnitude of any potential bias | Yes | Yes | Yes | Yes | No | No | No | Yes | No | No | No |
| Give a cautious overall interpretation of results considering objectives, limitations, multiplicity of analyses, results from similar studies, and other relevant evidence | Yes | Yes | Yes | Yes | No | No | No | Yes | No | No | No |
| Discuss the generalizability (external validity) of the study results | Yes | Yes | Yes | No | Yes | No | No | No | No | No | No |
| Funding—Give the source of funding and the role of the funders for the present study and, if applicable, for the original study on which the present article is based (b) Conﬂicts of interest— Describe any conﬂicts of interest, or lack thereof, for each author (c) Describe the authors’ roles— Provision of an authors’ declaration of transparency is recommended (d) Ethical approval—Include information on ethical approval for use of animal and human subjects (e) Quality standards—Describe any quality standards used in the conduct of the research | Yes | Yes | Yes | No | No | Yes | Yes | No | Yes | Yes | No |
| **Overall quality assessment score** | **17/22** | **19/22** | **18/22** | **16/22** | **14/22** | **11/22** | **11/22** | **16/22** | **15/22** | **13/22** | **13/22** |

# **Table 3 Quality assessment: The ARRIVE guidelines for reporting animal comparative studies (Kilkenny et al. 2010)**

| **Item** | **Gruen et al. 2017** | **Klinck et al. 2018a** | **Klinck et al. 2018b** |
| --- | --- | --- | --- |
| Provide as accurate and concise a description of the content of the article as possible. | Yes | Yes | Yes |
| Provide an accurate summary of the background, research objectives (including details of the species or  strain of animal used), key methods, principal findings, and conclusions of the study. | Yes | Yes | Yes |
| Include sufficient scientific background (including relevant references to previous work) to understand the motivation and context for the study, and explain the experimental approach and rationale; Explain how and why the animal species and model being used can address the scientific objectives, and where appropriate the study’s relevance to human biology. | Yes | Yes | Yes |
| Clearly describe the primary and any secondary objectives of the study, or specific hypotheses being tested. | Yes | Yes | Yes |
| Indicate the nature of the ethical review permissions, relevant licences (e.g. Animal [Scientific Procedures] Act 1986), and national or institutional guidelines for the care and use of animals, that cover the research. | Yes | Yes | Yes |
| For each experiment, give brief details of the study design, including: The number of experimental and control groups; Any steps taken to minimise the effects of subjective bias when allocating animals to treatment (e.g., randomisation procedure) and when assessing results (e.g., if done, describe who was blinded and when); The experimental unit (e.g. a single animal, group, or cage of animals). A time-line diagram or flow chart can be useful to illustrate how complex study designs were carried out. | Yes | Yes | Yes |
| For each experiment and each experimental group, including controls, provide precise details of all procedures carried out. For example: a. How (e.g., drug formulation and dose, site and route of administration, anaesthesia and analgesia used [including monitoring], surgical procedure, method of euthanasia). Provide details of any specialist equipment used, including supplier(s). When (e.g., time of day). Where (e.g., home cage, laboratory, water maze). d. Why (e.g., rationale for choice of specific anaesthetic, route of administration, drug dose used) | Yes | Yes | Yes |
| Provide details of the animals used, including species, strain, sex, developmental stage (e.g., mean or median age plus age range), and weight (e.g., mean or median weight plus weight range); Provide further relevant information such as the source of animals, international strain nomenclature, genetic modification status (e.g. knock-out or transgenic), genotype, health/immune status, drug- or test naı've, previous procedures, etc. | Yes | Yes | Yes |
| Provide details of: a. Housing (e.g., type of facility, e.g., specific pathogen free (SPF); type of cage or housing; bedding material; number of cage companions; tank shape and material etc. for fish). b. Husbandry conditions (e.g., breeding programme, light/dark cycle, temperature, quality of water etc. for fish, type of food, access to food and water, environmental enrichment). c. Welfare-related assessments and interventions that were carried out before, during, or after the experiment. | Yes | Yes | Yes |
| Specify the total number of animals used in each experiment and the number of animals in each experimental group; Explain how the number of animals was decided. Provide details of any sample size calculation used; Indicate the number of independent replications of each experiment, if relevant. | Yes | Yes | Yes |
| Give full details of how animals were allocated to experimental groups, including randomisation or matching if done; Describe the order in which the animals in the different experimental groups were treated and assessed. | Yes | Yes | Yes |
| Clearly define the primary and secondary experimental outcomes assessed (e.g., cell death, molecular markers, behavioural changes). | Yes | Yes | Yes |
| Provide details of the statistical methods used for each analysis. b. Specify the unit of analysis for each dataset (e.g. single animal, group of animals, single neuron). c. Describe any methods used to assess whether the data met the assumptions of the statistical approach. | Yes | Yes | Yes |
| For each experimental group, report relevant characteristics and health status of animals (e.g., weight, microbiological status, and drug- or test-naïve) before treatment or testing (this information can often be tabulated). | Yes | Yes | Yes |
| a. Report the number of animals in each group included in each analysis. Report absolute numbers (e.g. 10/20, not 50%a). b. If any animals or data were not included in the analysis, explain why. | Yes | Yes | Yes |
| Report the results for each analysis carried out, with a measure of precision (e.g., standard error or confidence interval). | Yes | Yes | Yes |
| a. Give details of all important adverse events in each experimental group. b. Describe any modifications to the experimental protocols made to reduce adverse events. | No | No | No |
| a. Interpret the results, taking into account the study objectives and hypotheses, current theory, and other relevant studies in the literature. b. Comment on the study limitations including any potential sources of bias, any limitations of the animal model, and the imprecision associated with the results a. c. Describe any implications of your experimental methods or findings for the replacement, refinement, or reduction (the 3Rs) of the use of animals in research. | Yes | Yes | Yes |
| Comment on whether, and how, the findings of this study are likely to translate to other species or systems, including any relevance to human biology. | No | No | No |
| List all funding sources (including grant number) and the role of the funder(s) in the study. | Yes | Yes | Yes |
| **Overall quality assessment score** | **18/20** | **18/20** | **18/20** |
